# The potential for integrated care programmes to improve quality of care as assessed by patients with COPD: early results from a real-world implementation study in The Netherlands

**DOI:** 10.5334/ijic.836

**Published:** 2012-09-18

**Authors:** Jane Murray Cramm, Maureen PMH Rutten-Van Mölken, Anna Petra Nieboer

**Affiliations:** Institute of Health Policy and Management (iBMG), Erasmus University, Rotterdam, The Netherlands; Institute of Health Policy and Management (iBMG), Erasmus University, Rotterdam, The Netherlands; Institute of Health Policy and Management (iBMG), Erasmus University, Rotterdam, The Netherlands

**Keywords:** chronic care, integrated care, disease management, COPD, chronic care model

## Abstract

**Objective:**

We investigated whether patients with chronic obstructive pulmonary disease (COPD) who were enrolled in disease-management programmes (DMPs) felt that they received a better quality of care than non-enrolled COPD patients.

**Methods:**

Our cross-sectional study was performed among patients (n=665) enrolled in four DMPs in the Netherlands. We also evaluated COPD patients (n=227) not enrolled in such programmes. Patients’ assessment of chronic-illness care (PACIC) was measured with a 20-item questionnaire. The instrument had five pre-defined domains: patient activation (three items), delivery-system/practice design (three items), goal setting/tailoring (five items), problem solving/contextual (four items), and follow-up/coordination (five items).

**Results:**

The mean overall PACIC score (scale: 1–5) of enrolled DMP patients was 2.94, and that of non-enrolled DMP patients was 2.73 (p≤0.01). Differences in the same direction were found in the subscales of patient activation (p≤0.01), delivery-system/practice design (p≤0.001), and problem solving/contextual (p≤0.001).

**Conclusions:**

Our results suggest that even in the early stages of implementation, DMPs for COPD may significantly improve care.

## Introduction

Chronic obstructive pulmonary disease (COPD) is a preventable and treatable disease characterised by progressive and incompletely reversible airflow limitation [1]. COPD is the leading cause of death from lung disease worldwide and the fourth leading cause of death overall [2], substantially contributing to the volume of emergency department visits and hospitalisations [3]. Early- and late-stage COPD remains under-diagnosed and under-treated [3, 4]. Historically, the medical community has focused on acute care and short-term goals that emphasise the management of acute exacerbations and complications and the reduction of recovery time. The ‘acute care model’ directs responsibility for problem solving to the clinician and responsibility for daily chronic-care management to the patient, typically without self-management support. A comprehensive approach to chronic-care management calls for multidisciplinary teams (e.g. nurses, therapists, social workers, pharmacists, dieticians) to support the individual over time and take responsibility for patient outcomes with general practitioners (GPs). Improved health status and outcomes have resulted from holistic and patient-centred programmes that offer self-management support services [5]. The literature strongly suggests that change may only be achieved through multicomponent interventions [6–8]. Integrated care models, such as disease-management programmes (DMPs), capture the complexity of coordinated healthcare provision for chronic conditions. DMPs aim to improve the effectiveness and economic efficiency of chronic-care delivery [9] by combining patient-related, professional, and organisational interventions [10].

Currently, the extent to which DMPs provide patients with better experiences and higher quality of care remains unclear. Assuming that the rationale underlying DMPs (i.e. evidence-based, structured care focused on patient activation) is legitimate and favours better outcomes, we should expect healthcare professionals participating in DMPs to provide higher-quality patient care than non-participating providers [11–13], and expect patients to perceive their care as such.

The Netherlands has implemented several parallel policies that target specific elements of the chronic-care continuum. To facilitate regulated competition, Dutch authorities have taken measures to improve information on healthcare quality, thereby supporting negotiations between healthcare purchasers and providers. Such measures have resulted in the development of a care standard for COPD that includes multidisciplinary evidence-based guidelines, process protocols, and performance indicators. Patients were involved in developing the measures to ensure patient empowerment within the context of regulated competition [14]. Furthermore, several health care reforms and a national health insurance system were implemented to stimulate integration of care [15, 16]. The introduction of the Health Insurance Act (Zvw) in 2006 was the most important, which set the foundations for a regulated market in the Dutch health care system [17].

The Dutch policies as well as most DMPs are based on the chronic care model (CCM) introduced by Edward Wagner [8, 18, 19]. The CCM was developed as a foundation for redesigning primary-care practices and forms the basis for effective chronic care management. It addresses shortcomings in acute care models by identifying essential elements that encourage high-quality chronic-disease care [8, 19] The model provides an organised multidisciplinary approach to the delivery of care for patients with chronic diseases, which involves the community and healthcare system and fosters communication between clinicians and well-informed patients. The goals of the CCM model are: (1) to improve chronic-disease management, (2) to facilitate the prevention of complications, and (3) to improve outcomes, including healthcare utilisation and quality of life.

The CCM has received widespread attention [20], including a recent evaluation of the elements that most improve care processes, costs, and clinical outcomes for patients with diabetes [21, 22]. The Patient Assessment of Chronic Illness Care (PACIC) instrument has been proven to be a reliable and valid tool [23] to measure quality of care according to the CCM. It addresses the extent to which provided care complies with the CCM from the patient’s perspective based on his or her experience. Because DMPs seek to structure care in accordance with the core elements of the CCM, we hypothesised that DMP-enrolled patients would achieve higher PACIC scores than non-enrolled patients, suggesting better quality of care. Specifically, we sought to determine whether patients with COPD who were enrolled in DMPs perceived quality of care to be better than patients with COPD who received regular care.

## Methods

### Study population

Our cross-sectional study included COPD patients recently enrolled in four newly implemented DMPs in various regions in the Netherlands (Tilburg, Arnhem, Monnickendam, and Almere). These DMPs are initiated and controlled by the practices. A national programme on ‘disease management of chronic diseases’ carried out by ZonMw (Netherlands Organisation for Health Research and Development) and commissioned by the Dutch Ministry of Health, provided funding for practices planning a redesigning of primary care for COPD patients according to the CCM. Requirements of the national programme were that the practices had to have some experience with the delivery of chronic care and were equipped to implement all systems needed for the delivery of sufficient chronic care, which resulted in the inclusion of these four DMPs for COPD patients. COPD patients not enrolled in DMPs were also included in the study sample. Randomisations of patients did not take place. Patients not enrolled in DMPs received regular care from GPs in 20 healthcare practices in the Netherlands. In addition, these healthcare practices implemented the COPD care standard but they did not implement a combination of patient-related, professionally-directed, and organisational interventions and are therefore not considered a DMP. The DMPs did implement a combination of patient-related, professionally-directed, and organisational interventions.

### The disease management programme

The DMP includes everyone who is diagnosed with COPD (based on GOLD criteria) no additional inclusion criteria were applied. The DMPs comprised a variety of collaborations—primarily GPs, physiotherapists, and dieticians—undergoing internal practice redesign to improve effective chronic-care management in 38 primary care practices. The DMPs, initiated and controlled by the practices, addressed shortcomings in acute care models by identifying essential elements that fostered high-quality chronic-disease care. We evaluated the four DMPs in their early stages to enhance our knowledge of disease-management experiments in chronic care and to stimulate successful programme implementation [24]. All DMPs use a combination of patient-related, professionally-directed, and organisational interventions. The key-interventions for patients are ‘action plans’, which provide education about the causes and symptoms of COPD, suggest ways to control symptoms and maintain physical functioning, train patients in the early recognition of acute exacerbations of COPD (AECOPD), and provide guidelines for developing treatment plans with self-initiated prescriptions. The DMP also offers patients non-medical interventions, such as smoking cessation support, nutrition therapy, and physical activity enhancement. Key-interventions for professionals are implementation of protocols, guidelines, COPD care standard and providing audit and feedback on performance indicators (e.g. lung function, exacerbation parameters and quality of life). Organisational key-interventions are implementation of a (regional) chain-integrated information system, implementation of (care standard) protocols in all information systems, distribution of hospital to primary care and delegation of care from General Practitioner (GP) to practice nurse, which makes the practice nurse the main care-coordinator in most disease management programs.

### Procedure

The patients were handed questionnaires during consultations or received them by mail. A reminder and a copy of the questionnaire were sent to non-respondents a few weeks later. Because the professionals were often time-strapped with implementation duties, they in some cases received hands-on support to administer questionnaires. The study was approved by the ethics committee of the Erasmus University Medical Centre of Rotterdam in September 2009. Data were collected anonymously and treated confidentially to protect sensitive patient information.

### Measures

Patients’ assessments of care were measured with the 20-item PACIC questionnaire, which used a five-point response scale ranging from ‘almost never’ to ‘almost always’ [23]. Higher scores represented a more frequent presence of the respective aspect of structured chronic care. The instrument had five pre-defined domains: patient activation (three items), delivery-system/practice design (three items), goal setting/tailoring (five items), problem solving/contextual (four items), and follow-up/coordination (five items) (see Appendix).

Basic demographic data on age, gender, marital status, self-rated health and educational level were also gathered. Educational level was dichotomised into ‘low’ and ‘high’, with low representing no or only some primary/secondary education.

### Statistical analyses

The PACIC was scored by summing each participant’s responses to all 20 items, then dividing by 20, the number of items in the scale. Missing values were replaced by mean scale scores if respondents filled in at least 2/3 of the items of a scale. Scores thus ranged from 1 to 5, with higher scores indicating the patients’ perception of greater involvement in self-management and receipt of chronic care counseling [23].

Differences between DMP/non-DMP patient groups were established with *t*-tests. Two-sided significance tests were conducted for differences between groups in background characteristics (Table 1) and PACIC overall and subscale scores (Table 2). All statistical analyses were conducted with SPSS software (version 17.0).

## Results

Table 1 provides the sample characteristics for DMP-enrolled (n=665) and non-DMP-enrolled (n=227) COPD patients. Of the 892 respondents (out of 1654; 54% overall response rate; of which 50% response rate in the control group and 58% response rate in the intervention group), 46% were female, 50% had a lower educational level, and 33% were single. Mean age was 66.1±10.6 years (range: 20–92 years). We found no significant differences in patient characteristics between groups; the two patient groups (patients in DMPs and patients who received regular care) are comparable in age, gender, marital status, educational level and self-rated health.

The mean overall PACIC score of DMP patients was 2.94, significantly higher than non-DMP patients (2.73; p≤0.01; Table 2). Differences in the same direction were found in the subscales of patient activation (p≤0.01), delivery-system/practice design (p≤0.001), and problem solving/contextual (p≤0.001). Mean scores for goal setting/tailoring (2.75 vs. 2.65) and follow-up/coordination (2.23 vs. 2.17) were higher for DMP patients, but not significantly. The greatest nominal difference was for problem solving (0.36) and the smallest was for follow-up/coordination (0.06).

## Discussion

In comparison with non-DMP-enrolees, patients enrolled in DMPs were more likely to receive CCM-directed, patient-centred, structured collaborative care. Our large cross-sectional study demonstrated significant differences in quality of care as assessed by the PACIC instrument. The differences were largest for the patient activation, delivery-system/practice design, and problem solving/contextual scales. Patients not participating in DMPs received lower levels of quality of care aspects measured by all subscales of the PACIC, albeit not all differences were statistically significant. In line with earlier research findings for diabetes patients enrolled in DMPs, DMP patients with COPD received better care than those who did not participate in such programmes [25, 26]. The higher but not significantly different mean scores of DMP patients for the goal setting/tailoring and follow-up/coordination subscales may be due to the early stage of DMP implementation. These two aspects of care typically provide benefits in the long-term, and we expect them to show significant improvement in future measurements. Other qualitative research also showed that a holistic approach is needed in the treatment of COPD, which includes an individual treatment plan aimed at smoking cessation, optimisation of pulmonary status by pharmacotherapy and exercise embedded in a new lifestyle [27].

The mean age of our study population is comparable to a study of Wensing and colleagues [28] who also studied PACIC scores among COPD patients in rural areas in the Netherlands. The mean age of COPD patients in their study population (n=77) was 67.2±11.7. Their study population contained fewer women (35%). They reported a mean overall PACIC score of 2.3 for COPD patients in 2008. Their COPD patients were recruited from general healthcare practices who did not implement a DMP or interventions to enhance structured/integrated care. The control practices in our study did implement the COPD care standard, which may explain the higher PACIC scores among both patient groups.

The most important limitation of this study is the cross-sectional nature of the data. Our findings will be updated and expanded when the results of the final evaluation of the national study become available. In particular, we will assess whether clinical parameters improve among DMP patients. Furthermore, the control practices were in the process of implementing interventions, such as the care standard that may have biased the measure of usual care (patients not enrolled in DMPs). We were also unable to determine whether improvements in quality of care delivery were caused by the DMPs or other factors, such as the implemented parallel policies that target specific elements of the chronic-care continuum in the Netherlands. A longitudinal study is necessary to establish whether implementation of a DMP leads to improved care for COPD patients on top of implementation of the COPD care standard or implementation of other innovations, such as self-management programmes or electronic patient record devices (e-health) [29]. In addition, future research is necessary investigating the effectiveness of DMPs for specific subgroups (e.g. high versus low educated/mild COPD versus severe COPD). To our knowledge, this is the first large study assessing different types of care (DMP versus non-DMP) in accordance with the CCM from COPD patients’ perspectives with the PACIC instrument. The evaluation of large-scale programmes during implementation was challenging. DMP professionals faced many barriers that delayed their implementation; a shift toward patient-centeredness and increased self-management support placed new demands on them and their organisations.

## Conclusion

Our results suggest that DMPs for COPD as currently established in primary-care settings in the Netherlands may significantly improve care. Patients recognised the changes in daily practice induced by the DMPs as care that was more structured and reflected the core elements of the CCM to a greater extent than regular care. The differences in patients’ perceptions may influence clinical and economic outcomes [22], since integrated care programmes based on the CCM that offer self-management support services have been shown to improve health status and outcomes [5, 30]. What makes this finding particularly interesting is that earlier studies evaluating the implementation of CCM elements assessed provider structures or addressed process parameters. Our results contribute valuable insight to the on-going discussion that seeks to identify effective improvements in the quality of care for patients with chronic conditions. Our findings suggest that COPD care may be improved through the implementation of integrated DMPs.

## Reviewers

**Stephen Abbott**, Research Fellow, Division of Health Services Management and Research, School of Health Sciences, City University London, Northampton Square, London EC1V 0HB, UK

**Birthe Dinesen**, Associate Professor, PhD, Master in Administration, SMI, Department of Health Science and Technology, Aalborg University, Denmark

**Diego A. Rodríguez**, MD, Respiratory Medicine Specialist, Barcelona, Spain

**Nina Szczygiel**, PhD Candidate, GOVCOPP-DEGEI, University of Aveiro, Portugal

## Figures and Tables

**Table 1.** Patient characteristics

	DMP n=665	Non-DMP n=227	p
Mean age (years)	65.9±10.4	66.5±11.2	0.519
Female subjects	46%	47%	0.859
Single (not married/living in partnership)	33%	33%	0.967
Low educational level	50%	49%	0.737
Self-rated health (0–100)	69.8±13.6	68.4±14.5	0.235

Data are expressed as means±standard deviation or n (%). DMP=disease-management programme.

**Table 2.** Mean Patient Assessment of Chronic-Illness Care (PACIC) scores (overall and subscales) of patients in disease-management programmes (DMPs) and patients who received regular care

	DMP n=665	Non-DMP n=227	p
Overall PACIC	2.94±0.88	2.73±0.94	0.004
Patient activation	3.06±1.16	2.79±1.23	0.006
Delivery-system/practice design	3.62±0.99	3.35±1.10	≤0.0001
Goal setting/tailoring	2.75±0.96	2.65±0.97	0.176
Problem solving/contextual	3.00±1.14	2.64±1.15	≤0.0001
Follow-up/coordination	2.23±0.94	2.17±1.02	0.433

Data are expressed as means±standard deviation.

## Appendix ‘Patient Assessment of Chronic Illness Care (PACIC) questionnaire’

When I received care for my chronic illness over the past 6 months, I was:

1. Asked for my ideas when we made a treatment plan
2. Given choices about treatment to think about
3. Asked to talk about any problems with my medicines or their effects
4. Given a written list of things I should do to improve my health
5. Satisfied that my care was well organized
6. Shown how what I did to take care of my illness influenced my condition
7. Asked to talk about my goals in caring for my illness
8. Helped to set specific goals to improve my eating or exercise
9. Given a copy of my treatment plan
10. Encouraged to go to a specific group or class to help me cope with my illness
11. Asked questions, either directly or on a survey, about my health habits
12. Sure that my doctor or nurse thought about my values and my traditions when they recommended treatment to me
13. Helped to make a treatment plan that I could do in my daily life
14. Helped to plan ahead so I could take care of my illness even in hard times
15. Asked how my chronic condition affects my life
16. Contacted after a visit to see how things were going
17. Encouraged to attend programs in the community that could help me
18. Referred to a dietician, health educator or counsellor
19. Told with other types of doctors, like the eye doctor or surgeon, helped my treatment
20. Asked how my visits with other doctors were going

Patient activation: items 1–3; Delivery-system/practice design: items 4–6; Goal setting/tailoring: 7–11; Problem solving/contextual: 12–15; Follow-up/coordination:16–20.
